# Development and validation of the isothermal recombinase polymerase amplification assays for rapid detection of *Mycoplasma ovipneumoniae* in sheep

**DOI:** 10.1186/s12917-020-02387-3

**Published:** 2020-06-01

**Authors:** Jinfeng Wang, Ruiwen Li, Xiaoxia Sun, Libing Liu, Xuepiao Hao, Jianchang Wang, Wanzhe Yuan

**Affiliations:** 1 Technology Center of Shijiazhuang Customs District, Shijiazhuang, 050051 China; 2 College of Veterinary Medicine, Hebei Agricultural University, No.2596 Lekai South Street, Baoding, Hebei 071001 People’s Republic of China; 3 Hebei Academy of Science and Technology for Inspection and Quarantine, Shijiazhuang, 050051 China

**Keywords:** *Mycoplasma ovipneumoniae*, 16S rRNA gene, Real-time RPA, Lateral flow strip, Isothermal amplification

## Abstract

**Background:**

Mycoplasmal pneumonia is an important infectious disease that threatens sheep and goat production worldwide, and *Mycoplasma ovipneumoniae* is one of major etiological agent causing mycoplasmal pneumonia. Recombinase polymerase amplification (RPA) is an isothermal nucleic acid amplification technique, and RPA-based diagnostic assays have been described for the detection of different types of pathogens.

**Results:**

The RPA assays using real-time fluorescence detection (real-time RPA) and lateral flow strip detection (LFS RPA) were developed to detect *M. ovipneumoniae* targeting a conserved region of the 16S rRNA gene. Real-time RPA was performed in a portable florescence scanner at 39 °C for 20 min. LFS RPA was performed in a portable metal bath incubator at 39 °C for 15 min, and the amplicons were visualized with the naked eyes within 5 min on the lateral flow strip. Both assays were highly specific for *M. ovipneumoniae*, as there were no cross-reactions with other microorganisms tested, especially the pathogens involved in respiratory complex and other mycoplasmas frequently identified in ruminant*s*. The limit of detection of LFS RPA assay was 1.0 × 10^1^ copies per reaction using a recombinant plasmid containing target gene as template, which is 10 times lower than the limit of detection of the real-time RPA and real-time PCR assays. The RPA assays were further validated on 111 clinical sheep nasal swab and fresh lung samples, and *M. ovipneumoniae* DNA was detected in 29 samples in the real-time RPA, 31 samples in the LFS RPA and 32 samples in the real-time PCR assay. Compared to real-time PCR, the real-time RPA and LFS RPA showed diagnostic specificity of 100 and 98.73%, diagnostic sensitivity of 90.63 and 93.75%, and a kappa coefficient of 0.932 and 0.934, respectively.

**Conclusions:**

The developed real-time RPA and LFS RPA assays provide the attractive and promising tools for rapid, convenient and reliable detection of *M. ovipneumoniae* in sheep, especially in resource-limited settings. However, the effectiveness of the developed RPA assays in the detection of *M. ovipneumoniae* in goats needs to be further validated.

## Background

*Mycoplasma ovipneumoniae* is one of the major pathogens that cause mycoplasma pneumonia in sheep, goats, and wild ruminants [1–5]. *M. ovipneumoniae*-associated respiratory disease is characterized by cough, gasp, runny noses, progressive weight loss, pulmonary interstitial hyperplasia inflammation, and variable morbidity and mortality rates between flocks [6, 7]. Moreover, upon *M. ovipneumoniae* infection, sheep and goats become susceptible to other common pathogens causing respiratory disease, such as *Mannheimia haemolytica*, *Pasteurella multocida* and Parainfluenza-3 virus [8, 9]. Since first confirmed in Australia in 1972, infections by *M. ovipneumoniae* have been an endemic problem worldwide and have caused severe economic losses to the sheep and goat industry [10–12].

Bacteriological culture of *M. ovipneumoniae* is currently the gold standard for diagnosis, however, the culture is cumbersome and time-consuming due to the fastidious nature of the bacterium as well as that the follows required species identification by biochemical or serological tests, which make the assay burdensome for the routine applications [13–15]. In addition, the bacterial isolation may be hampered by sample contamination and prior antibiotic treatments received by the diseased animals. Serological tests, such as ELISA, indirect hemagglutination assay, are the common and economic methods for *M. ovipneumoniae* herd surveillance [12, 16]. However, seroconversion to *M. ovipneumoniae* is often delayed after natural infection, which makes the serology less effective in detecting early-stages of infection in herds, and unsuitable for detecting acute mycoplasmal pneumonia in the field [9, 14]. It is an urgent need to develop a rapid and accurate method to detect *M. ovipneumoniae*. Different nucleic acid amplification-based methods have been described to be sensitive and specific for *M. ovipneumoniae*, i.e. PCR, real-time PCR, and loop-mediated isothermal amplification (LAMP) [9, 14, 15]. PCR assays require a well-equipped laboratory, expensive equipment and trained personnel, which limits their application in the under-equipped laboratories and the point-of-need (PON) diagnosis [9, 15]. Compared to the PCR assays, the isothermal amplification methods have advantages regarding convenience to perform and minimal equipment requirement. A LAMP assay for the detection of *M. ovipneumoniae* has been described for low requirement of experimental conditions, however, the assay requires 60 min to complete the reaction [14].

Recombinase polymerase amplification (RPA), an isothermal DNA amplification technique, is rapid, reliable and considered to be a promising approach for PON diagnosis [17, 18]. RPA-based diagnostic assays have been described for the detection of different pathogens from different clinical samples [19, 20]. In this study, a real-time RPA assay using the exo probe and a LFS RPA assay using the nfo probe combined with lateral flow strip were developed for rapid, specific and sensitive detection of *M. ovipneumoniae*. The performance of the assays was further assessed by collecting and detecting the clinical sheep nasal swab and lung samples.

## Results

### Analytical specificity and sensitivity of the RPA assays

**Fig. 1:**
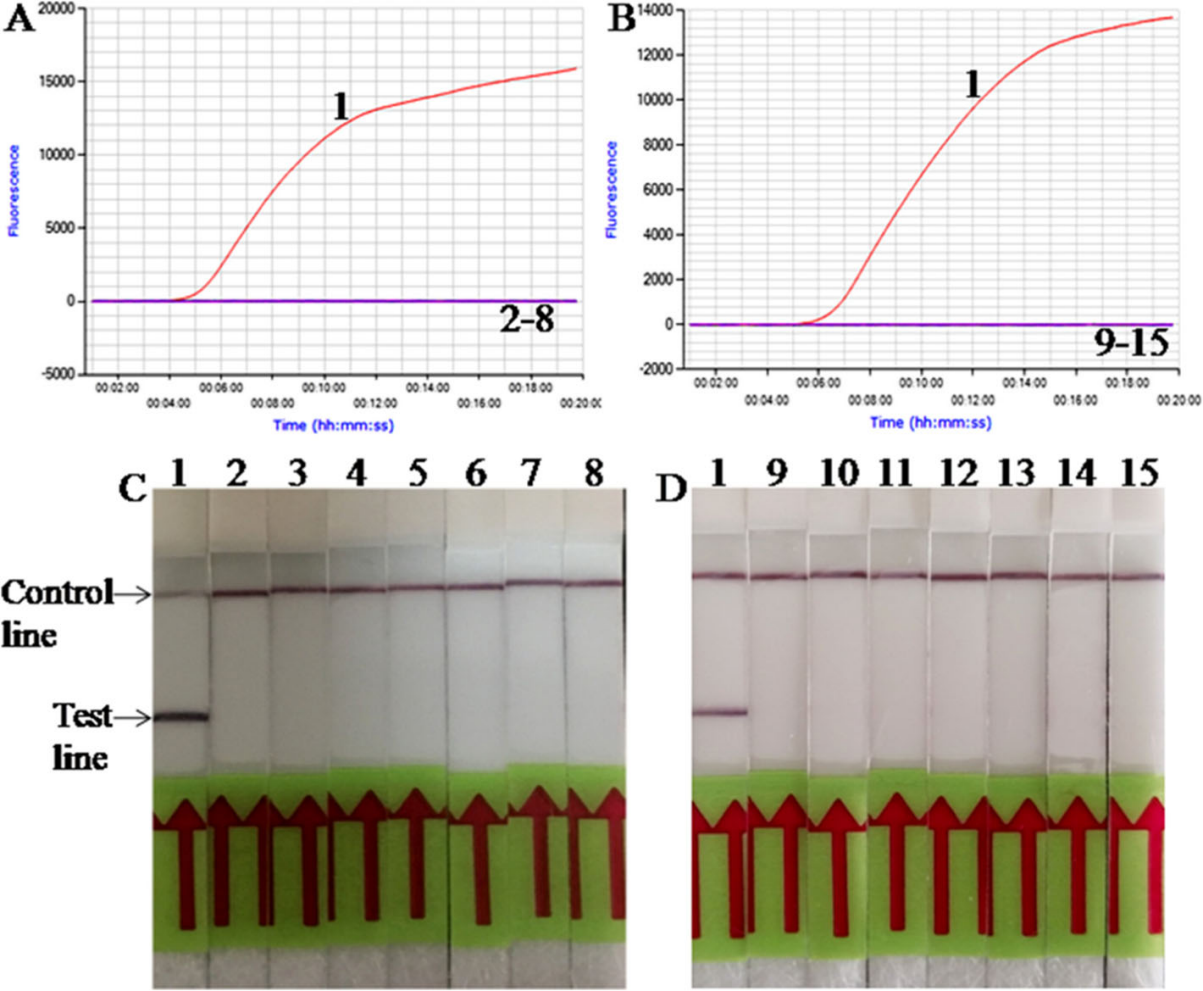
Analytical Specificity of *M. ovipneumoniae* real-time RPA (**a**, **b**) and LFS RPA (**c**, **d**) assays. Only the *M. ovipneumoniae* was amplified, but not other pathogens tested (*n* = 5). lane 1, *M. ovipneumoniae*; lane 2, *M. capricolum subsp. capripneumoniae*; lane 3, *M. mycoides subsp. capri*; lane 4, *M. arginini*; lane 5, *M. agalactiae*; lane 6, *P. multocida*; lane 7, *K. pneumoniae*; lane 8, PPRV; lane 9, *M. bovis*; lane 10, *M. flocculare*; lane 11, *M.bovoculi*; lane 12, *M.leachii*; lane 13, *M. capricolum subsp. capricolum*; lane 14, *M.dispar*; lane 15, *M. haemolytica*

Only *M. ovipneumoniae* was amplified in both real-time RPA and LFS RPA assays (Fig. 1). The specificity analysis was repeated five times with similar results, which demonstrated the good repeatability of the RPA assays.

**Fig. 2:**
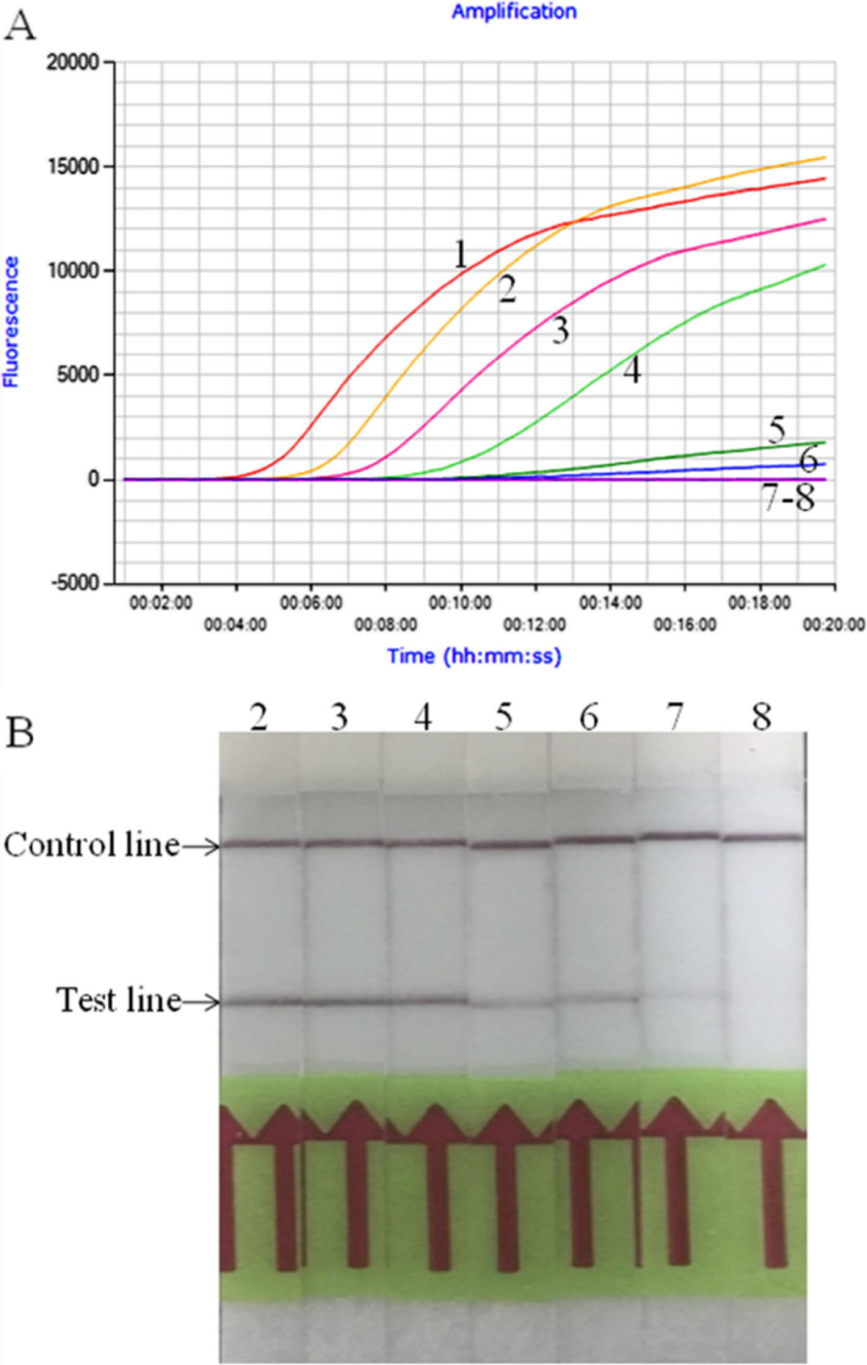
Analytical Sensitivity of *M. ovipneumoniae* real-time RPA (**a**) and LFS RPA (**b**) assays. The LOD of the real-time RPA was 1.0 × 10^2^ copies per reaction of *M. ovipneumoniae* standard DNA, while the LOD of the LFS RPA was 1.0 × 10^1^ copies per reaction. Lane 1, 1.0 × 10^7^ copies; lane 2, 1.0 × 10^6^ copies; lane 3, 1.0 × 10^5^ copies; lane 4, 1.0 × 10^4^ copies; lane 5, 1.0 × 10^3^ copies; lane 6, 1.0 × 10^2^ copies; lane 7, 1.0 × 10^1^ copies; lane 8, 1.0 × 10^0^ copies

**Fig. 3:**
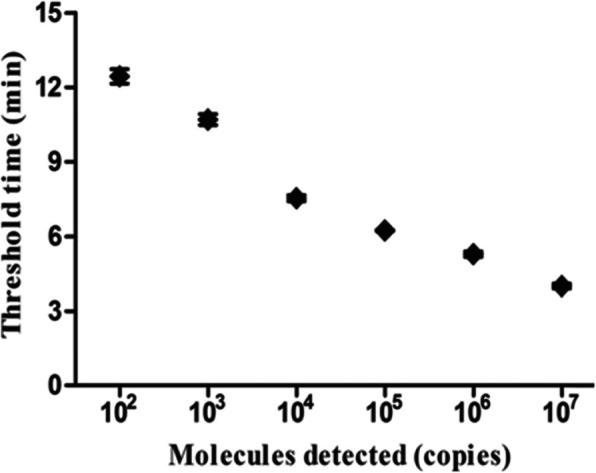
Reproducibility of *M. ovipneumoniae* real-time RPA assay. The analytical sensitivity was determined on DNA molecular standard (8 runs) for real-time RPA. Semi-logarithmic regression of the data collected from real-time RPA test runs on the DNA molecular standards using Prism Software. The run time of the real-time RPA was between 4 min–13 min for 1.0 × 10^7^–1.0 × 10^2^ copies *M. ovipneumoniae* standard DNA

The limit of detection of LFS RPA assay was 1.0 × 10^1^ copies *M. ovipneumoniae* standard DNA per reaction (Fig. 2a), while the LOD of real-time RPA was 1.0 × 10^2^ copies per reaction (Fig. 2b), which was same as that of the real-time PCR (data not shown). The real-time RPA assay was further performed eight times on the standard DNA, and 1.0 × 10^7^–1.0 × 10^2^ copies DNA molecules were detected in 8/8 runs, 1.0 × 10^1^ -1.0 × 10^0^, 0/8, which demonstrated the good reproducibility (Fig. 3).

### Validation of the RPA assays on clinical samples

Of the 111 sheep clinical samples, *M. ovipneumoniae* DNA was detected in 29 (26.12%), 31 (27.93%) and 32 (28.83%) samples by the real-time RPA, LFS RPA and real-time PCR, respectively (Table 1). Compared to the real-time PCR assay, the real-time RPA assay and LFS RPA assay showed diagnostic specificity (DSp) of 100 and 98.73%, diagnostic sensitivity (DSe) of 90.63 and 93.75%, positive predictive value (PPV) of 100 and 96.77%, negative predictive value (NPV) of 96.34 and 97.5%, and kappa value of 0.932 and 0.934, respectively (Table 2). The real-time RPA and LFS RPA assays demonstrated the comparable performance in detecting the 111 sheep clinical samples. In the RPA assays, it took no more than 20 min to obtain the positive results, while it need approximately 32 min - 46 min in the real-time PCR with the Ct values ranging from 20.77 to 36.52.

## Discussion

The developed real-time RPA and LFS RPA assays are highly specific and sensitive for detection of *M. ovipneumoniae* in the sheep clinical samples. Both RPA assays performed well at 39 °C within 20 min, which is faster than other common nucleic acid amplification methods. The real-time RPA assay and LFS RPA assay were performed on the tube scanner Genie III and a metal bath incubator, respectively. These two pieces of equipment are portable, lightweight, easily carried and can be charged by battery for working a whole day. For the RPA reagents, they are provided in the form of lyophilized powder and are independent of cold chains. Several studies also demonstrated that RPA was tolerant to most of the PCR inhibitors [19, 21]. The above characteristics make the developed RPA assays ideal for the detection of *M. ovipneumoniae* in field which is especially important for farms located in rural areas.

The PCR and LAMP assays targeted on the 16S rRNA gene, elongation factor TU gene or adhesin P113 gene had demonstrated their efficacy in the detection of *M. ovipneumoniae* in different clinical specimens, including the nasal swabs and lung samples [4, 9, 14, 15]. The RPA primers and probes were designed basing on the 16S rRNA gene of *M. ovipneumoniae* in this study. To ensure that the target sequences were unique to *M. ovipneumoniae*, we screened the selected primers and probes in silico using the pattern searching tool function from the EMBOSS package against the genomes of the common mycoplasmas causing infections in ruminants [22]. The complementary regions could not be found when allowing 1 or 5 sequence mismatches for the primer sequences. Furthermore, there was no mismatch in the reverse primers and probes in the *M. ovipneumoniae* strains available in Genbank, and only one mismatch in the forward primer in two strains: 2013–12,928-46 (Accession number: MN028079) and NCTC10151 (Accession number: LR215028.1). According to the above in silico analysis, the designed primers and probes fulfilled the specificity requirements of RPA [23]. In the specificity analysis, both the real-time RPA and LFS RPA only amplified the genomic DNA of *M. ovipneumoniae*, and no other mycoplasmas, bacteria and PPRV. Most importantly, *M. capricolum* subsp. *capripneumoniae*, the etiological agent of contagious caprine pleuropneumonia, was not amplified by the new developed RPA assays. Although the in silico sequence analysis support that all the *M. ovipneumoniae* strains are detectable, more genomic DNA of different strains of *M. ovipneumoniae* should be tested for further confirmation.

With the real-time PCR as the reference assay, the diagnostic performances of the developed real-time RPA and LFS RPA assays were evaluated. The performances of the RPA assays were comparable to the real-time PCR, while the RPA assays were faster to obtain the detection results. Furthermore, the developed real-time RPA was slightly weak in the detection of the clinical samples containing low amounts of *M. ovipneumoniae* DNA, as three nasal samples were negative in real-time RPA assay while positive in real-time PCR with Ct values of 36.49, 35.50 and 36.52. The above results are inspiring, but the RPA assays should be further validated on more clinical samples, especially those containing low amounts of *M. ovipneumoniae* DNA.

## Conclusions

In this study, we describe the development of the real-time RPA and LFS RPA assays for the simple, rapid and reliable detection of *M. ovipneumoniae* from the sheep nasal and lung samples. The developed RPA assays could be performed in field conditions without the need of any expensive equipment, and could also become a routine test for rapid and direct detection of *M. ovipneumoniae* in the farm.

## Methods

### Bacteria, virus strains, clinical samples and DNA extraction

Genomic DNA of *M. ovipneumoniae* (Y98) and genomic DNA or cDNA of a panel of pathogens involved in respiratory complex and other mycoplasmas frequently identified in ruminant were maintained in our laboratory and used in the study, which were the following 6 mycoplasmas, 3 non-mycoplasma bacteria and 1 virus: *M. capricolum subsp. capripneumoniae* (F38), M. *mycoides subsp. capri* (PG3), *M. arginini* (G230), *M. agalactiae* (PG2), *M. bovis* (PG45), *M. flocculare* (HB-XS3), *Mannheimia haemolytica* (F120G3), *Klebsiella pneumoniae* (F21W3), *Pasteurella multocida* (F91G3) and *Peste des petits ruminants viru*s (Nigeria 75/1 vaccine strain). Four artificial constructs, pUC57-Mbovoculi, pUC57-Mleachii, pUC57-Mcc and pUC57-Mdispar, were also used in the study. The constructs contain the full 16S rRNA gene of *M.bovoculi* (1531 bp), *M.leachii* (1524 bp), *M. capricolum subsp. capricolum* (1466 bp) and *M.dispar* (1475 bp), which were synthesized artificially by Sangon Biotech (Shanghai, China) based on the reference sequences available in GenBank (Accession numbers: CP007154, NR_044773, NR_118796, NR_025182).

A total of 46 sheep clinical samples (30 nasal swabs and 16 fresh lungs) were collected in Baoding City, Hebei Province from October to November 2019. The nasal swabs were collected from the sheep with coughing symptom in Fangzhuang farm in Dingzhou County, Baoding City, and the sheep fresh lungs were obtained from Zhuanluzhen slaughter house in Tang County, Baoding City. The sheep nasal swabs and lung samples were treated and the total DNA was extracted as described previously [24]. Furthermore, 65 nucleic acid samples extracted from the clinically healthy sheep nasal swabs were kindly provided by Dr. Qingan Han from Hebei Animal Disease Prevention and Control Center. The 65 sheep nasal swabs were collected in October–December 2019, in which 35 samples were collected from one sheep farm in Tang Country, Baoding City and the other 30 samples were collected from one sheep farm in Pingquan County, Chengde City. All the samples were used for the daily sheep disease surveillance. The 65 nucleic acid samples were also quantified using a ND-2000c spectrophotometer (NanoDrop, Wilmington, USA) and used in the study.

### Generation of standard DNA

To generate a *M. ovipneumoniae* standard DNA for the RPA assays, a PCR product containing 361 bp covering the region of interest of 16S rRNA gene was amplified from the *M. ovipneumoniae* DNA using LMF1 and LMR1 as primers (Table 3) and cloned into the pMD19-T (Takara, Dalian, China) for standards. The resulting plasmid, pMO-16SrRNA, was transformed into *Escherichia coli* DH5α cells, purified with the SanPrep Plasmid MiniPrep Kit (Sangon Biotech, Shanghai, China) and quantified. The copy number of DNA molecules was calculated by the following formula: amount (copies/μL) = [DNA concentration (g/μL)/ (plasmid length in base pairs× 660)] × 6.02 × 10^23^. Ten-fold dilutions of the pMO-16SrRNA, ranging from 1.0 × 10^7^ to 1.0 × 10^0^copies/μL, were prepared in nuclease-free water and aliquots of each dilution were stored at − 80 °C.

**Table 1:** Comparison of *M. ovipneumoniae* real-time RPA, LFS RPA and real-time PCR assays for detection of clinical samples

Origin	Location	Sample	Number	Real-time RPA		LFS RPA		Real-time PCR	
				P	N	P	N	P	N
Farm 1	Dingzhou County, Baoding	nasal swabs	30	11	19	11	19	13	17
Farm 2	Tang County, Baoding	nasal swabs	35	4	31	5	30	5	30
Farm 3	Pingquan County, Chengde	nasal swabs	30	8	22	9	21	8	22
Slaughter house	Tang County, Baoding	fresh lungs	16	6	10	6	10	6	10
		T	111	29	82	31	80	32	79

**Table 2:** Diagnostic sensitivity, diagnostic specificity, predictive value, and kappa value of real-time RPA, LFS RPA and real-time PCR assays for diagnosing *M. ovipneumoniae* infection

	real-time PCR		
	P	N	T
real-time RPA			
P	29	0	29
N	3	79	82
T	32	79	111
	DSe:90.63%	DSp:100%	K:0.932
	PPV:100%	NPV:96.34%	
LFS RPA			
P	30	1	31
N	2	78	80
T	32	79	111
	DSe:93.75%	DSp:98.73%	K:0.934
	PPV:96.77%	NPV:97.5%	

*P* positive, *N* negative, *DSe* diagnostic sensitivity, *DSp* diagnostic specificity, *K* kappa value, *PPV* positive predictive value, *NPV* negative predictive value

**Table 3:** Sequences of the primers and probes for *M .ovipneumoniae* real-time RPA, LFS RPA and PCR assays

Assay	Primers and probes	Sequence 5′-3′	Amplicon size (bp)	References
real-time RPA	MO-exo-F	TGAGTAACACGTACCTAACCTACCTTTTGGAC	254	This study
	MO-exo-R	TGCTGCCTCCCGTAGGAGTCTGGGCCGTATCTC		
	MO-exo-P	TTGGTAGGGTAAAGGCCTACCAAGACGATGA (FAM-dT)(THF)(BHQ1-dT)TTAGCGGGGCCAAGAG-C3-spacer		
LFS RPA	MO-nfo-F	TGAGTAACACGTACCTAACCTACCTTTTGGAC	254	This study
	MO-nfo-R	Biotin-TGCTGCCTCCCGTAGGAGTCTGGGCCGTATCTC		
	MO-nfo-P	FAM-TTGGTAGGGTAAAGGCCTACCAAGACGATGAT(THF)TTTAGCGGGGCCAAGAG-C3-spacer		
real-time PCR	Mo16S_35F	TGGGTGAGTAACACGTACCTAACC	62	[4]
	Mo16S_96R	AGCCGCTGTTTCCAATGG		
	Mo16S_60T	FAM-ACCTTTTGGACCGGGATA-MGB		
PCR	LMF1	TGAACGGAATATGTTAGCTT	361	[9]
	LMR1	GACTTCATCCTGCACTCTGT		

### RPA primers and probe

The 16S rRNA gene of *M. ovipneumoniae* was determined as the amplification target for RPA. According to the reference sequences of *M. ovipneumoniae* (Accession numbers: NR_025989.1, LR215028.1, MN028361, MN028184, MN028079, MH133233), the highly conserved region in the 16S rRNA gene was identified, and the RPA primers, exo and nfo probes were designed following the RPA manufacturer guidelines (TwistDx. Cambridge, UK). Primers and probe are listed in Table 3 and synthesized by a commercial company (Sangon Biotech, Shanghai, China).

### Real-time RPA and LFS RPA assays

The *M. ovipneumoniae* real-time RPA assay was performed as described previously [24]. The total reaction volume was 50 μL including 40.9 μL of Buffer A (rehydration buffer), 2.0 μL of each RPA primers (MO-exo-F and MO-exo-R, 10 μmol/L), 0.6 μL of exo probe (MO-exo-P, 10 μmol/L) and 2.5 μL of Buffer B (magnesium acetate, 280 mmol/L). Furthermore, 1 μL of genomic DNA or recombinant plasmid was used for the specificity and sensitivity analysis, or 2 μL of sample DNA was used for the clinical sample diagnosis.

The *M. ovipneumoniae* LFS RPA assay was also performed as described previously [24]. The total reaction volume was 50 μL including 29.5 μL of rehydration buffer, 2.1 μL of each RPA primers (MO-nfo-F and MO-nfo-R, 10 μmol/L), 0.6 μL of exo probe (MO-nfo-P, 10 μmol/L) and 2.5 μL of magnesium acetate (280 mmol/L). In addition, 1 μL of bacterial genomic DNA or recombinant plasmid was used for the specific and sensitive analysis, or 2 μL of sample DNA was used for the clinical sample diagnosis. The assay was performed in a metal bath incubator at 39 °C for 15 min. Furthermore, the lateral flow strips (Milenia Biotec GmbH, Germany) were used to detect the RPA amplicons dual-labeled with FAM and biotin.

### Analytical specificity and sensitivity analysis

Both RPA assays were performed to amplify the nucleic acids of a panel of microorganisms including *M. ovipneumoniae*, *M. capricolum subsp. capripneumoniae*, *M. mycoides subsp. capricolum*, *M. arginini*, *M. agalactiae*, *M.bovoculi*, *M.leachii*, *M. capricolum subsp. capricolum*, *M.dispar*, *M. bovis, M. flocculare*, *M. haemolytica*, *P. multocida, K. pneumoniae*, PPRV, which are considered to be dangerous to the sheep and goat respiratory system or frequently identified in the ruminants. The analytical specificity analysis was repeated five times.

The standard DNA of *M. ovipneumoniae*, ranging from 1.0 × 10^7^ to 1.0 × 10^0^ copies/μL, was used for the RPA analytical sensitivity analysis. One microliter of each dilution was amplified by both RPA assays to determine the limit of detection (LOD). The analytical sensitivity analysis was repeated five times. Furthermore, the real-time RPA was tested using the standard DNA in 8 replicates, the threshold time was plotted against the molecules detected and a semi-log regression was calculated using Prism software 5.0 (Graphpad Software Inc., SanDiego, California).

### Validation with clinical samples

The RPA assays were validated with 95 sheep nasal swabs and 16 sheep fresh lungs. All samples tested with the two RPA assays were also tested by a real-time PCR in parallel. The real-time PCR for *M. oviopneumoniae* was performed on a ABI 7500 instrument (Applied Biosystems, Foster City, California), which was described previously [4].

## Data Availability

The dataset analyzed during the current study is available from the corresponding author on reasonable request. The nucleotide sequences under the relevant accession numbers (CP007154, NR_044773, NR_118796, NR_025182, NR_025989.1, LR215028.1, MN028361, MN028184, MN028079, MH133233, MN028079, LR215028.1) analysed during the current study are available in the GenBank repository, https://www.ncbi.nlm.nih.gov/nuccore/.

## Abbreviations

CT: Cycle threshold
DSp: Diagnostic specificity
DSe: Diagnostic sensitivity
ELISA: Enzyme linked immunosorbent assay
K. pneumonia: *Klebsiella pneumoniae*
LAMP: Loop-mediated isothermal amplification
LFS: Lateral flow strip
M. agalactiae: Mycoplasma agalactiae
M. arginini: Mycoplasma arginini
*M. bovis*: Mycoplasma bovis
M.bovoculi: Mycoplasma bovoculi
M. capricolum subsp. capripneumoniae: Mycoplasma capricolum subsp. capripneumoniae
M.dispar: Mycoplasma dispar
M. flocculare: Mycoplasma flocculare
M. haemolytica: Mannheimia haemolytica
M.leachii: Mycoplasma leachii
M. mycoides subsp. capri: Mycoplasma mycoides subsp. capri
M. ovipneumoniae: Mycoplasma ovipneumoniae
NPV: Negative predictive value
PCR: Polymerase chain reaction
*P. multocida*: *Pasteurella multocida*
PON: Point-of-need
PPRV: Peste des petits ruminants virus
PPV: Positive predictive value
RPA: Recombinase polymerase amplification
TT: Threshold time
